# Immunohistochemical Expression of Wnt-4 Protein in Clear Cell Renal Carcinoma

**DOI:** 10.3390/jcm10245795

**Published:** 2021-12-11

**Authors:** Oliver Pavlovic, Tvrtko Hudolin, Ivan Miskulin, Stela Bulimbasic, Marijana Coric, Josip Perkovic, Toni Zekulic

**Affiliations:** 1 Department of Urology, University Hospital Centre Osijek, 31000 Osijek, Croatia; oliver.pavlovic@mefos.hr (O.P.); jperkovic@gmail.com (J.P.); 2 Department of Surgery, Urology, Orthopedics and Physical and Rehabilitation Medicine, Faculty of Medicine Osijek, The Josip Juraj Strossmayer University of Osijek, 31000 Osijek, Croatia; 3 Department of Urology, University Hospital Centre Zagreb, 10000 Zagreb, Croatia; toni.zekulic@gmail.com; 4 Zagreb School of Medicine, University of Zagreb, 10000 Zagreb, Croatia; stela.bulimbasic@gmail.com (S.B.); marijanacoric17@gmail.com (M.C.); 5 Department of Public Health, Faculty of Medicine Osijek, The Josip Juraj Strossmayer University of Osijek, 31000 Osijek, Croatia; ivan.miskulin@mefos.hr; 6 Department of Pathology and Cytology, University Hospital Centre Zagreb, 10000 Zagreb, Croatia

**Keywords:** clear cell renal carcinoma, Wnt signaling pathway, Wnt-4 protein, immunohistochemistry

## Abstract

Wingless binding integration site proteins (Wnt) have an important role in normal kidney development and in various kidney diseases. They are required for complete epithelial differentiation and normal nephron formation. Changes in these proteins could also have important role in carcinogenesis. This study included 185 patients with clear cell renal carcinoma (ccRCC) in whom immunohistochemical expression of Wnt-4 protein in healthy and tumorous tissue after surgery was investigated. There was higher expression of Wnt-4 in healthy than in tumor tissue. No difference between Fuhrman’s grade and Wnt-4 expression was found. A poor negative correlation between tumor size and Wnt-4 expression was found. Patients with suspected metastatic diseases had higher Wnt-4 expression. There was no difference in survival rates between Wnt-4 negative and positive groups. In our study we have shown that high Wnt-4 expression in healthy tissue decreases in low-grade tumors but then increases in high-grade tumors, suggesting that tumor progression requires Wnt-4 activation or reactivation.

## 1. Introduction

Kidney cancer accounts for about 2–3% of cancers worldwide and is one of the most common urological malignancies, with more than 330,000 newly diagnosed cases per year, mostly in Europe, North America, Australia and Japan [1]. It is estimated that there are about 85,000 new cases of kidney cancer in the European Union each year, while about 35,000 patients die from the disease [2]. Due to the widespread use of imaging modalities such as ultrasound, computed tomography (CT) or magnetic resonance imaging, most patients with kidney cancer are now diagnosed at an earlier stage of the disease, with better treatment outcomes [3]. Renal cancer is a complex disease consisting of several subtypes, of which clear cell carcinoma (ccRCC) is the most common form that causes significant morbidity and mortality, so a better understanding of its biology is very important [4,5].

Normal kidney development is a complex process involving many molecules, and one of the most important is the family of wingless binding integration site proteins (Wnt) required for grouping cells for complete epithelial differentiation, i.e., nephron formation [6,7]. However, changes in Wnt proteins have also been shown in various types of tumors, such as hepatocellular carcinoma, thyroid cancer and head and neck squamous cell carcinoma, but also in adrenal gland tumors and kidney cancer [8,9]. In this study, we investigated the immunohistochemical expression of one member of the Wnt family, Wnt-4, which has been attributed the most important role not only in kidney development, but also in different kidney diseases [10]. The aim of this study was to determine Wnt-4 presence and its potential role as an indicator of ccRCC biological activity.

## 2. Materials and Methods

### 2.1. Patients

This retrospective cohort study included a group of 185 patients with ccRCC who underwent surgery at the Department of Urology, University Hospital Centre Zagreb, from September 2015 to April 2019. Patients did not have any prior therapy or known renal disease that could affect treatment outcome or Wnt-4 expression. Demographic and patient data including information about age, sex, CT findings, clinical stage, tumor location, type of surgery (partial vs. radical nephrectomy), as well as pathohistological results were collected, analyzed and correlated with Wnt-4 expression. Patients were followed up for at least one year after the surgery in accordance with European urological association (EUA) guidelines [11]. We used the 2017 TNM classification and Fuhrman’s grading system [11,12]. The study was approved by the Institutional Ethical Review Board (8.1-20/160-2).

### 2.2. Immunohistochemistry

**Figure 1:**
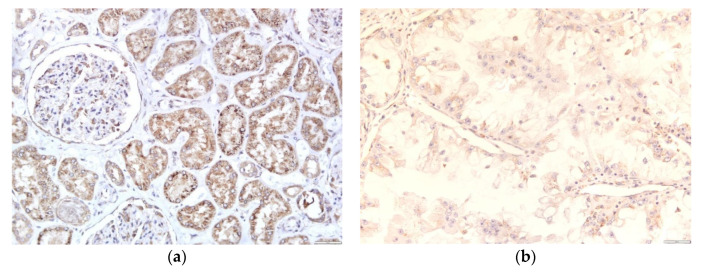

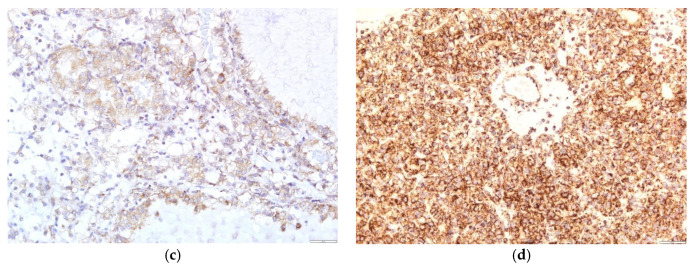
Immunohistochemical stain for Wnt-4: (**a**) strong and uniform cytoplasmic Wnt-4 positivity in tubular epithelial cells. Podocytes along the glomerular tuft and Bowman’s parietal epithelial cells are also positive for Wnt-4 (magnification 200×); (**b**) a weak cytoplasmic positivity in tumor cells for Wnt-4 (magnification 400×); (**c**) a moderate cytoplasmic staining of the tumor cells for Wnt-4 (magnification 400×); (**d**) diffuse, strong cytoplasmic staining for Wnt-4 of the tumor cells (magnification 200×).

The tissue was fixed in 10% buffered formalin, dehydrated in ascending order of alcohols, embedded in paraffin blocks, and cut to a thickness of 3–4 microns. Antigen unmasking was performed in TP-Link High Buffer pH 9.0 3-in-1. After unmasking, the tissue was incubated with the primary anti-WNT-4 (B-6) antibody Santa Cruz Biotechnology, Inc. (Santa Cruz Biotechnology, Inc., Dallas, Texas, U.S.A.) diluted 1:50, for 30 min at room temperature. After the incubation with the primary antibody, samples were incubated for 10 min with a buffer-washed peroxidase blocking reagent, and the tissue was incubated with the EnVision FLEX/HRP secondary antibody (Agilent, Santa Clara, California, U.S.A.) for 30 min. The entire staining procedure was done in Autostainer Link 48 (Agilent, Santa Clara, California, U.S.A.). Immunostaining was semi-quantitatively evaluated for intensity (0 = negative; 1(+) = weak; 2(++) = moderate; and 3(+++) = strong staining). (Figure 1). For detecting Wnt-4 expression in healthy tissue we used macroscopically and histologically healthy kidney tissue from the 35 patients.

### 2.3. Statistical Analysis

Statistical processing and analysis of the data were performed using the program STATISTICA 6.1 (StatSoft Inc., Tulsa, Oklahoma, USA). Patient demographic data were described by descriptive statistics (numerical data) and frequency tables (descriptive data). Comparison of Wnt-4 expression in macroscopically and microscopically healthy tissue and in tumor tissue was performed with a t-test for independent samples. Comparison of Wnt-4 and tumor grade and comparison of Wnt-4 and tumor stages were performed using analysis of variance (ANOVA) and Fisher LSD test. Comparison of Wnt-4 and TNM groups, subgroups, and suspected metastases was performed using a t-test for independent samples. The relationship between Wnt-4 and the biggest diameter of the tumor size was analyzed by correlation. Multivariate Cox regression and Kaplan–Meier curve were used in survival analysis. The statistical differences between several different groups of patients were tested by the chi-square test.

## 3. Results

A total of 185 patients were included in the study, 69 (37.3%) women and 116 (62.7%) men. The mean age of the patients was 60.2 years (34–83). In 108 (58.4%) patients, the tumor was on the right kidney. Regarding location, in 72 (38.9%) patients the tumor was on the upper pole, in 62 (33.5%) in the middle, and in 51 (27.6%) in the lower pole of the kidney. Six (3.2%) patients had suspected metastatic disease in lungs, three (1.6%) in the liver, twelve (6.5%) in lymph nodes, nine (5%) in a suprarenal gland and four (2.2%) in bones. Radical nephrectomy was performed in 127 (68.6%) and partial nephrectomy in 58 (31.35%) patients. The mean tumor size was 5.32 cm (1–19). Nine (4.9%) patients had Fuhrman grade 1, 108 (58.4%) grade 2, 53 (28.6%) grade 3 and 15 (8.1%) had grade 4. Tumorous infiltration of the renal capsule was found in 30 (16.2%), of the renal vein in 46 (24.9%) and the vena cava was infiltrated in 4 (2.2%) patients. Adipose tissue was infiltrated in 56 (30.3%) patients, and 6 (3.2%) patients had positive lymph nodes, while in 109 (58.9%) patients lymphadenectomy was not performed. Adrenalectomy was performed in 40 (21.6%) patients, of which 5 were metastatic. A total of 111 (60%) patients had T1, 8 (4.3%) T2, 64 (34.6%) T3 and 2 (1.1%) T4 disease stages. All the data are reported in Table 1.

**Figure 2:**
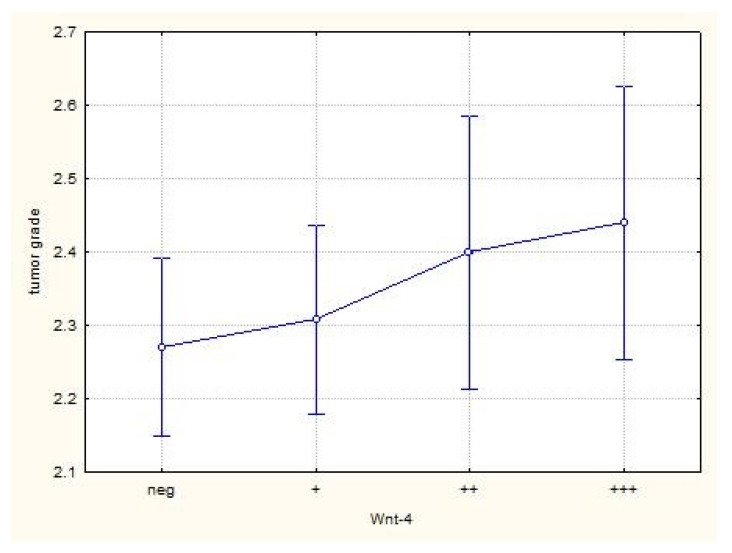
Correlation of Wnt-4 expression and tumor grade. Due to the small number of patients, grades 1 and 4 are excluded (neg; no immunohistochemical staining, (+) weak; (++) moderate; (+++) strong immunohistochemical staining).

**Figure 3:**
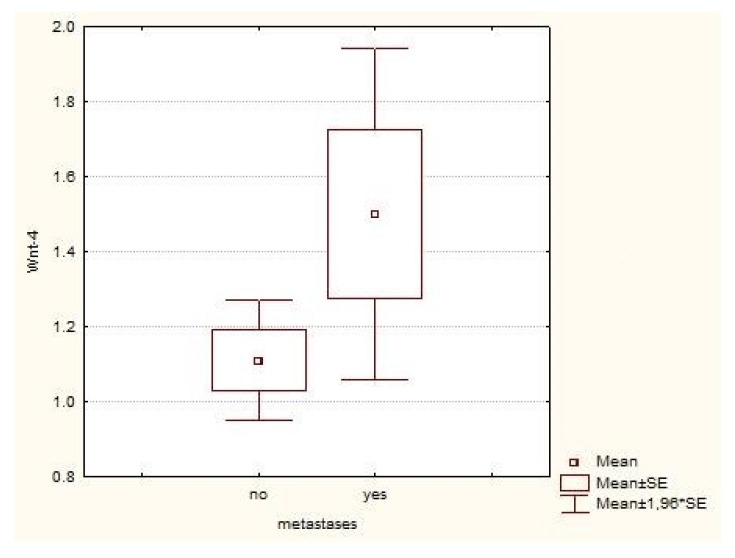
Wnt-4 expression in patients with and without metastases. There is a difference between the groups, but due to the large difference in the number of patients with and without metastases it is not significant (SE; standard error).

Wnt-4 expression in healthy tissue was high, on average 2.8 (0–3), and in tumor tissue on average 1.2 (1–3) (*p* <0.001, statistical significance at a significance level of 99% (α = 0.01)) (Table 2). Most of our patients had grade 2 or 3 tumors, and a proportional correlation between Wnt-4 positivity and increasing grade was found (*p* = 0.403), but without statistically significant difference (Figure 2). Approximately equal Wnt-4 expression (1.18 vs. 1.17) was observed when comparing pathological stages T1 and T3 (since there were only eight T2 and two T4 patients, they were excluded from the analysis) (*p* = 0.929). The same results were reported for T1a vs. T1b and T3a vs. T3b subgroups. When we compared the tumor size in centimeters for all patients, there was a poor negative correlation between tumor size and Wnt-4 expression, i.e., larger tumors had less Wnt-4 expression (r = −0.1240, *p* = 0.093, statistical significance at a significance level of 90% (α = 0.1)). Patients with suspected metastatic disease had higher mean Wnt-4 expression (1.5 vs. 1.1) compared with patients without metastases, but without significant differences between them (*p* = 0.104) (Figure 3).

**Figure 4:**
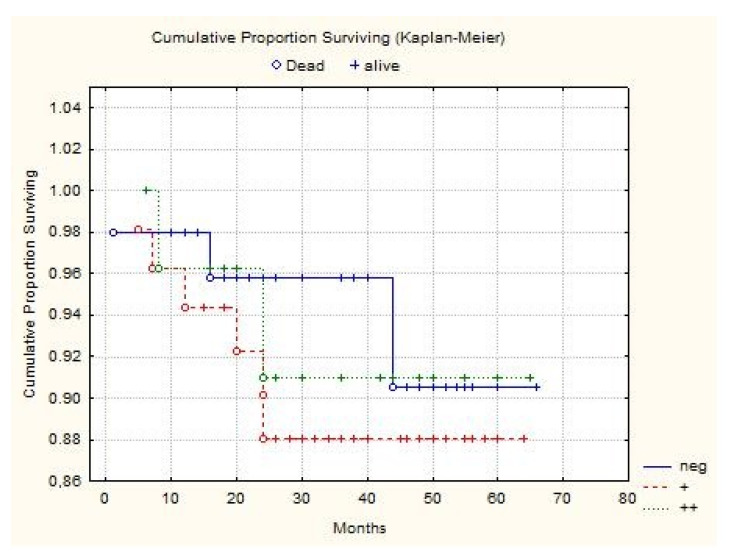
Cumulative proportion surviving of Wnt-4 negative and Wnt-4 positive groups.

The average follow-up time was 33 months (12–60). There was no difference in survival rates between Wnt-4 negative and Wnt-4 positive groups (*p* = 0.578) (Figure 4).

## 4. Discussion

Highly conserved Wnt genes encode a large group of 19 proteins involved in different cellular processes, from development and normal function to carcinogenesis. They are necessary for the grouping of kidney progenitor cells, followed by complete epithelial differentiation, i.e., nephron formation, as shown by Wnt-4 expression in developing kidneys [6,7,13]. However, high Wnt-4 expression is also present in normal kidney tissue (mostly in proximal tubules). In our study, we have also shown that 80% of our macroscopically and microscopically healthy tissue had high Wnt-4 expression, showing that the canonical Wnt pathway is necessary for adult kidney homeostasis as proposed by Lancaster et al. [14].

In many cases, carcinogenesis uses different developmental processes, so it was logical to investigate the expression of Wnt-4, which has already been shown to play an important role in various renal diseases. Furthermore, a high expression found in normal epithelium of proximal tubules from which ccRCC arises was an additional reason for investigation [15]. Our cohort of ccRCC can be divided into three groups: first, without any expression; second, with weak expression; and the third group with medium to high Wnt-4 expression. When we compared healthy tissue with ccRCC, a statistically significant reduction of Wnt-4 expression in ccRCC was found. It has already been shown that different tumor suppressors such as p63 and p73 positively regulate Wnt-4 expression, suggesting that its appropriate maintenance could be critical for adult tissue homeostasis and prevention against tumor initiation [16,17], but this may change once the tumor progresses to more aggressive forms.

A significant number of our cancer samples had high levels of Wnt-4 expressions. When the correlation between Wnt-4 expression and well-known parameters for ccRCC prognosis, such as tumor grade and stage were made, we found conflicting results. Since we had only 9 patients with Fuhrman’s grade 1 and 15 patients with grade 4 tumors, they were excluded from the analysis. For the remaining patients, no statistically significant correlation between grade and Wnt-4 expression was found, but a continuous increase of mean positivity with increasing grades was noticed. Although not statistically significant, it shows that Wnt-4 expression is maintained, moreover, it continuously increases with increasing tumor grade. It has already been shown that Wnt-4 secreted by tumor tissue promotes cancer progression. Furthermore, elevated levels of Wnt-4 in serum are downregulated after tumor resection [18]. In our patients with suspected metastatic diseases, there was increased Wnt-4 expression, and, although this was not statistically significant, it suggests that ccRCC preserves this pathway for further development. It has also been shown that the alteration in Wnt signaling is important for two distinctive features of the tumor population: therapeutic resistance and immune escape, which are markers of tumor aggression [19]. As with some other signaling molecules that have been shown to have several different, and—in some cases depending on different circumstances—quite opposite functions, we can only confirm that Wnt signaling is not a binary system whereby the pathway is on or off and has a single unique activity, but rather is highly regulated in a compound fashion to provide spatial and temporal variation depending on multiple environmental factors [20,21].

Since Wnt signaling is important not only for tumor development and progression, but also for the immune response, different strategies and a range of drugs/molecules targeting different components of this pathway have been investigated [22]. Some of them have shown anticancer effects, mainly on breast and colorectal cancer, even synergistic effects in combination with chemotherapy, but have not yet been approved for clinical use. On the other hand, some other drugs already used for other diseases, such as niclosamide, nigericin or psoralen, target and inhibit Wnt signaling in multiple cancers [23,24,25]. This approach can save time and costs, but since Wnt signaling is important for normal development and homeostasis, there is also the issue of side-effects that are likely to be overcome by more specific cancer targeting. Whether these drugs will have a place in the treatment of patients with kidney cancer, whether this treatment will be affected by Wnt-4 expression, and whether the drugs should be used alone or in combination with some other novel treatment options for metastatic renal cancer remains to be determined [26,27]. The ongoing preclinical/clinical trials could define the role of the Wnt pathway in different therapeutic areas and hopefully open new opportunities for patients with kidney cancer [28].

We have investigated a group of ccRCC patients with different clinical and pathohistological findings which make our group heterogeneous and therefore hard to draw strong, unambiguous conclusions from. What we also need to consider is heterogeneity within the tumor itself, which can cause different levels of expression within the same tumor samples. Nevertheless, we have shown that high Wnt-4 expression in healthy tissue decreases in low-grade tumors, but then increases in high-grade tumors, suggesting that tumor progression requires Wnt-4 activation or reactivation. Since most of our patients had stage 1 and 3 diseases with a relatively short follow-up period, we can only speculate about the possible role of Wnt-4 expression as a marker for the survival of patients with ccRCC. Relatively short follow-up, single-institution study, and immunohistochemistry alone that did not include multiple areas from the same patient/tumor to address tumor heterogeneity can be considered as possible weaknesses of this study. Furthermore, we had a low number of high-grade ccRCC patients in whom different Wnt-4 expression can be expected, but this may be explained by the significant stage reduction caused by the systemic use of different imaging modalities, which has allowed for early diagnosis of ccRCC. The inclusion of preclinical research as well as more molecular methods and samples would increase the quality of this study.

Wnt-4 expression in ccRCC as well as in different kidney diseases is complex and not unambiguous. If years of research have taught us one thing, it is that there is nothing basic and simple about this pathway. Further studies are needed to elucidate the role of Wnt-4 protein in ccRCC.

## Figures and Tables

**Table 1:** Patients’ descriptive data.

	Median (Range)
Age	60.2 (34–83)
Tumor size	5.32 (1–19)
	Number of patients (percentage)
Total number of patients Female Male	185 69 (37.3) 116 (62.7)
Left kidney Right kidney	108 (58.4) 77 (41.6)
Tumor location Upper Middle Lower	72 (38.9) 62 (33.5) 51 (27.6)
Total nephrectomy Partial nephrectomy	127 (68.6) 58 (31.35)
Fuhrman’s grade I II III IV	9 (4.9) 108 (58.4) 53 (28.6) 15 (8.1)
TNM T1 T2 T3 T4	111 (60.0) 8 (4.3) 64 (34.6) 2 (1.1)
Patients with suspected metastatic disease on MSCT or X-rays	22 (11.9)

TNM: Classification of Malignant Tumors (tumor, node, metastasis); T1–T4: primary tumor stage 1–4; MSCT: multi-slice computed tomography.

**Table 2:** Wnt-4 expression in micro and macroscopically healthy tissue and in ccRCC.

Wnt-4	Healthy Tissue	ccRCC
Negative	0	61 (33%)
Positive	35 (18.9)	124 (67%)
(+) (++) (+++)	1 (2.6%) 6 (17.1%) 28 (80.3%	63 (34.1%) 32 (17.3%) 29 (15.7%)

Wnt-4: Wingless binding integration site protein—4; ccRCC: clear-cell renal carcinoma; (+) weak; (++) moderate; (+++) strong immunohistochemical staining.

## Data Availability

The data presented in this study are available on request from the corresponding author. The data are not publicly available due to privacy and ethical reasons.
